# Histone Methylation of H3K4 Involved in the Anorexia of Carnivorous Mandarin Fish (*Siniperca chuatsi*) After Feeding on a Carbohydrate-Rich Diet

**DOI:** 10.3389/fendo.2020.00323

**Published:** 2020-06-19

**Authors:** Jun-Jie You, Ping Ren, Shan He, Xu-Fang Liang, Qian-Qian Xiao, Yan-Peng Zhang

**Affiliations:** ^1^College of Fisheries, Chinese Perch Research Center, Huazhong Agricultural University, Wuhan, China; ^2^Innovation Base for Chinese Perch Breeding, Key Lab of Freshwater Animal Breeding, Ministry of Agriculture, Wuhan, China

**Keywords:** mandarin fish, carbohydrate-rich diets, transcriptome sequencing, H3K4 tri-methylation, food intake, anorexia

## Abstract

Food intake of carnivorous fish decreases after feeding on a carbohydrate-rich diet. However, the molecular mechanism underlying the anorexia caused by high-carbohydrate diets has remained elusive. We domesticated the mandarin fish to feed on carbohydrate-rich (8%) diets. After 61 days of feeding, several fish (Group A) fed well on artificial diets during the whole feeding period; the other fish (Group B) fed well on artificial diets at the beginning of the feeding period, with their food intake then decreasing to half (anorexia) and then to zero for 5 days; and, finally, a negative control (Group C) fed on live prey fish throughout the experimental process. The plasma glucose was significantly higher in the mandarin fish of Group B than in those of Group A, whereas levels of hepatic glycogen and plasma triglyceride were significantly lower. Using transcriptome sequencing, we investigated the differentially expressed genes between Groups A and B and excluded the genes that were not differentially expressed between Groups A and C. The activation of mTOR and Jak/STAT pathways were found in the mandarin fish with anorexia, which was consistent with the higher expression levels of *pepck* and *pomc* genes. We found a higher expression of histone methyltransferase *setd1b* gene and an increased histone H3 tri-methylated at lysine 4 (H3K4me3) in the fish of Group B. Furthermore, using ChIP assay and inhibitor treatment, we found that the up-regulated H3K4me3 could activate *pepck* expression, which might have contributed to the hyperglycemia and anorexia in the mandarin fish that fed on carbohydrate-rich diets. Our study initially indicated a link between histone methylation and *pepck* expression, which might be a novel regulatory mechanism of fish who are fed a carbohydrate-rich diet.

## Introduction

In aquaculture, carbohydrates are added to artificial diets as the energy source to save protein (1, 2). However, several reports demonstrate the negative effects of carbohydrates on food intake (3, 4). Carbohydrates could be an efficient inhibitor of appetite; food intake decreases after feeding a carbohydrate-rich diet in mammals and carnivorous fish (4, 5). Previous research shows that hyperglycemia could contribute to a decreased food intake in mammals after feeding on high-carbohydrate diets (6–8). In rainbow trout, high-carbohydrate diets (10.5%) reduce the food intake by changing the parameters of glucose-sensing, such as the increased levels of glucose, glucose 6-phosphate, and glycogen (9, 10). In mammals, glucose is an important regulatory signal that controls the secretion of hormones by various endocrine cells and activates neurons in the nervous system (11). The regulation of food intake is mediated by a neuronal circuit that integrates orexigenic and anorexigenic signals such as NPY/AGRP and POMC/CART (12). However, little is known about the regulatory mechanisms of hyperglycemia on anorexia induced by high-carbohydrate diets in fish.

It has been noted that epigenetic regulation through histone modification may be shaped by environmental factors (13), such as nutritional factors consisting of protein and carbohydrates. Epigenetic modification has been observed in baboons with reduced nutrient availability (14). The abundance of histone H3K27me3 decreased in the kidneys of rats fed with high-fat diets (15). Histone modifications correlate with transcriptional activation and repression. Decreased H3K4me3 in oocytes caused the global down-regulation of transcription activity, and oocytes failed to complete maturation (16). Therefore, the regulative effect of histone modifications on food intake might contribute to the anorexia induced by carbohydrate-rich diets. SETD1B is a histone methyltransferase that specifically methylates “Lys-4” of histone H3. SETD1B tri-methylates “Lys-4” of histone H3 to regulate iNOS expression, and inhibition of SETD1B decreases H3K4me3 enrichment at the *NOS2* promoter region and diminishes iNOS expression (17). Most studies about SETD1B are mainly in the field of cancer and pathology in mammals, but there are little studies about its role in the glucose metabolism and food intake control.

The molecular mechanism underlying the anorexia caused by high-carbohydrate diets has remained elusive in fish, which is related to the poor utilization of dietary starch, especially in carnivorous species with lower glucose intolerance (18, 19). With mandarin fish, as a typical carnivorous fish, once their fry start feeding, they feed solely on the live fry of other fish species and refuse zooplankton or formulated diets (20). In the present study, we domesticated the mandarin fish (*Siniperca chuatsi*) to feed on the high-carbohydrate (8%) artificial diets and identified the fish with or without anorexia after feeding high-carbohydrate diets. We examined the biochemical indicators involved in glucose metabolism and the differentially expressed genes and pathways between the two groups based on transcriptome sequencing. In addition, Western blotting assay of histone methylation H3K4me3, treatment of histone methyltransferase SETD1B inhibitor, and chromatin immunoprecipitation (ChIP) assays of H3K4me3 and *phosphoenolpyruvate carboxykinase* (*pepck*) gene have allowed us to gain insights into the molecular mechanism of anorexia in mandarin fish after feeding on high-carbohydrate diets. It could support the development of low-cost artificial diets for carnivorous fish.

## Materials and Methods

### Reagents and Fish

Tri-Methyl-Histone H3 (lys4) (C42D8) Rabbit mAb and Phospho-S6 (Ser235/236) (D57.2.2E) XP® Rabbit mAb were obtained from Cell Signaling Technology (Danvers, USA). Goat anti-rabbit and goat anti-mouse second antibody were purchased from Bioss (Beijing, China). Mandarin fish were obtained from Chinese Perch Research Center of Huazhong Agricultural University (Wuhan, China). Fish (50 ± 5 g) were maintained in aquarium (60 × 45 × 45 cm, 9 tanks, 15 fish per tank) with a continuous system of water filtration and aeration at constant temperature (25 ± 0.5°C). The animal protocol was approved by the Institutional Animal Care and Use Ethics Committee of Huazhong Agricultural University (Wuhan, China).

Mandarin fish were domesticated to feed on the high-carbohydrate (8%) artificial diets (Table 1) following the domestication methods (21). After 61 days of feeding, several fish, named Group A, fed well on an artificial diet during the whole feeding period; the other fish, named Group B, fed well on artificial diet at the beginning, but the food intake decreased to half (anorexia: the mandarin fish of Group B ate <3 feed pellets, and fish of Group A ate 5–7 feed pellets), and did not even eat artificial diets for 5 days. Besides Group A and B, we also set a negative control named Group C, which fed with live prey fish through the experimental process. We carried out the experiment with three biological replicates (3 tanks for each group with 15 fish per tank). Before sampling, mandarin fish from each group were fed with live prey fish to eliminate the effect of hunger on the mRNA and protein expression levels. The fish were anesthetized with MS-222 (Argent Chemical Laboratories, Redmond, USA) (200 mg/L), and the blood was drawn from the tail vein. The liver and body samples were frozen in liquid nitrogen upon surgical resection and restored in a −80°C. The whole blood sample was 4,000 rpm for 10 min, collecting plasma and stored at −80°C.

**Table 1:** Composition of the high-carbohydrate diets.

**Ingredients**	**%**
White fish meal	71
Corn starch	8
Fish oil	10
Vitamin premix^1^	2
Mineral premix^2^	2
Microcrystalline cellulose	2
Carboxymethyl cellulose	2
Yeast extract powder	3

1 *Vitamin premix (per kg of diet): vitamin B1 (thiamin), 30 mg; vitamin B2 (riboflavin), 60 mg; vitamin B6, 30 mg; vitamin B12, 0.22 mg; vitamin D3, 5mg; vitamin E 160 mg; vitamin K3 50 mg; folic acid, 20 mg; biotin, 2.5 mg; pantothenic acid calcium, 100 mg; ascorbic acid (35%), 250 mg; niacinamide, 200 mg; powdered rice hulls, 999 mg*.

2 *Mineral premix (per kg of diet): MnSO4, 10 mg; MgSO4, 10 mg; KCl, 95 mg; NaCl, 165 mg; ZnSO4, 20 mg; KI, 1 mg; CuSO4, 12.5 mg; FeSO4, 105 mg; Na2SeO3, 0.1 mg; Co, 1.5 mg*.

### Nutrient Metabolite Assay

The assays of plasma glucose, hepatic and muscle glycogen, plasma triglyceride, and plasma insulin were performed with Glucose Assay Kit, Liver/Muscle Glycogen Assay Kit, Insulin Assay Kit, and Triglyceride Assay Kit (Nanjing Jiancheng Bioengineering Institute, China), respectively, according to the manufacturers instructions, with six biological replicates and three technical replicates.

### RNA Isolation and Reverse Transcription

Total RNA was extracted using Trizol reagent (TaKaRa, Tokyo, Japan) following the manufacturer's instructions. The extracted RNA was re-suspended in 30 μL RNase-free water and then quantified with a BioTek Synergy™ 2 Multi-Detection Microplate Reader (BioTek Instruments, Winooski, USA) and agarose gel electrophoresis. One microgram of total RNA was synthesized to complementary DNA (cDNA) using Revert Aid™ Reverse Transcriptase (TaKaRa, Tokyo, Japan) according to the manufacturer's instructions. The synthesized cDNA was stored at −20°C.

### Transcriptome Sequencing

Equal amount of total RNA from each group (three biological replicates for each group) were used to construct the libraries for transcriptome analysis using MGIEasy RNA kit following manufacturer's instructions (BGI, Wuhan, China). Purified Poly(A) mRNA was from total RNA via oligo-dT-attached magnetic beads. Paired-end cDNA libraries were sequenced using BGISEQ-500 system (BGI, Wuhan, China). SOAPnuke was used to perform image deconvolution and base calling. Clean reads were obtained by removing adaptor reads and low-quality reads (Q ≤ 10), on which all the following analyses were based. Transcriptome assembly was carried out with the short reads assembling program Trinity, with a k-mer length of 25 bp. The reads were mapped back to assembled contigs. By using the paired-end information, contigs, as well as the distances between these contigs, could be detected from the same transcript. We connected the contigs using N to represent unknown sequences between each pair of contigs, and then scaffolds were made. Paired-end reads were used again for filling the gaps of scaffolds to obtain sequences with the least amount of Ns and could not be extended on either end. Such sequences were defined as unigenes. To annotate the transcriptome, we performed the BLAST alignments between unigenes and databases, such as Kyoto Encyclopedia of Genes and Genomes (KEGG), Gene Ontology (GO), NR, NT, SwissProt, Pfam, and KOG, with Blast2GO, hmmscan, and getorf software.

To estimate expression levels, the RNA-Seq reads generated were mapped to the unigenes using Bowtie2. Gene expression levels were measured by RSEM. We analyzed the differentially expressed genes used DEGseq method described before (22), with a false discovery rate (FDR) of ≤0.001 and fold change of ≥2.00 as the threshold to judge the significance of gene expression difference. GO function and KEGG pathway analysis were then carried out for the differentially expressed genes.

### Real-Time Quantitative PCR

Primers were designed with Primer 5.0 software, based on the sequences that were obtained from transcriptome sequencing data of mandarin fish, and synthesized by Sangon (Shanghai, China) (Table 2). Several housekeeping genes, including *beta-actin, ywha2, b2m, rpl13a, hmbs*, and *sdha*, were selected according to the literature (23). *Rpl13a* gene was more stable and amplified as the internal control. Real-time quantitative PCR was carried out with MyiQ™ 2 Two-Color Real-Time PCR Detection System (Bio-Rad, Hercules, USA). PCR was performed in a total volume of 20 μL containing 10 μL AceQ® qPCR SYBR® Green Master Mix (Vazyme, Piscataway, USA), 8.2 μL RNase Free H_2_O, 0.4 M of each primer, and 1 μL cDNA. The PCR procedure parameters were 95°C for 5 min, followed by 40 cycles of 10 s at 95°C, with an annealing temperature for 30 s. Melt curve analysis was performed from 65–95°C, gradually increasing 0.5°C/6 s, to verify the specificity. Gene expression levels were quantified relative to the expression of *rpl13a* using the optimized comparative Ct (2^−ΔΔ*Ct*^) value method (24). Data were presented as mean ± SEM, with six biological replicates and three technical replicates.

**Table 2:** Nucleotide sequences of the primers.

**Primers for real-time PCR**	**Sequences (5^**′**^-3^**′**^)**
*rpl13a*-F	CACCCTATGACAAGAGGAAGC
*rpl13a*-R	TGTGCCAGACGCCCAAG
ChIP-*pepck*-F	TCAACTGGCAAAACGAA
ChIP-*pepck*-R	ACCACTGCTGGCACTATC
*pepck*-F	GTCGGCTGTCCTCTACCACTCA
*pepck*-R	CCTCCTCCTTGGCAATACGC

### Western Blot

Liver tissue stored at −80°C was solubilized in lysis buffer, and lysates were separated on 10% SDS-PAGE gel. Proteins were then transferred onto PVDF membrane. The protein level of phospho-S6 (p-S6) and H3K4me3 were detected by immunoblotting with the antibody (1:1,000–1:4,000). Blots were probed by goat anti- rabbit or goat anti-mouse second antibody with IR-Dye 680 or 800 cw, labeled (1:2,000–1:4,000; LiCor, Lincoln, USA) at room temperature for 1 h. The membranes were then visualized using a LiCor Odyssey scanner (Licor, Lincoln, USA) and quantified with ImageJ 1.44 software (National Institutes of Health, Bethesda, USA) with six biological replicates.

### ChIP Assay

A ChIP assay was performed using a SimpleChIP® Enzymatic ChIP kit (CST, USA). The liver tissues were isolated for DNA purification with ChIP Kit as described previously (25). Each DNA sample was divided into three parts: positive control (10% of each DNA sample), the experimental sample (45%), and negative control (45%). The positive control did not include the immunoprecipitation step; the experimental sample and negative control were analyzed by immunoprecipitation with specific H3K4me3 antibody (CST, USA; 1:50) and general rabbit IgG antibody (CST, USA; 1:125), respectively. After reverse crosslinking of Protein/DNA complexes, DNA was purified and used for ChIP-PCR to amplify the promoter regions of *pepck* gene. Real-time PCR was carried out in a 20 μL reaction mixture [10 μL AceQ® qPCR SYBR® Green Master Mix (Vazyme, Piscataway, USA), 0.4 μL of primers, 8.2 μL of distilled water, and 1 μL of DNA] by using a MyiQ™ 2 Two-Color Real-Time PCR Detection System (Bio-Rad, Hercules, USA) with the following conditions: 95°C for 5 min, followed by 40 cycles of 95°C for 10 s, with an annealing temperature for 30 s. Melt curve analysis was performed from 65°C to 95°C, gradually increasing 0.5°C/6 s, to verify the specificity. Reactions were performed in triplicate for each sample. The promoter primer sequences of *pepck* were 5′-TCAACTGGCAAAACGAA-3′ (forward primer) and 5′-ACCACTGCTGGCACTATC- 3′ (reverse primer), locating at −264 bp to −163 bp. The ChIP assay was performed with three biological replicates.

### Chaetocin Treatment

Hepatocytes were obtained from the liver tissue of mandarin fish. The liver tissue was cut into small pieces and digested with trypsin (Gibco, USA). Tissue was dispersed into cells through cell strainer (Biosharp, China). Red cells were lysed with red cell lysis buffer (Biosharp, China). Cells were cultured with M199 (10% fetal bovine serum, penicillin-streptomycin solution 1‰) (Gibco, USA). The inhibitor of SET1DB chaetocin (17) (Selleck, USA) was used to treat the cells at a concentration of 3 × 10^−5^ mol/L for 17 h, and then the levels of H3K4me3 and *pepck* mRNA expression were examined with six biological replicates and three technical replicates.

### Statistical Analysis

Statistical analyses were conducted with SPSS 19.0 software. All data were tested for normality and homogeneity of variances using the Shapiro-Wilk's test and Levene's test, respectively. One-way analysis of variance (ANOVA) was used to find significant differences, followed by Duncan's multiple range tests and Fisher's least-significant difference *post hoc* test, after confirming data normality and homogeneity of variances. Differences were considered to be significant if *P* < 0.05.

## Results

### Biochemical Indicators Related to Glucose Metabolism

**Figure 1:**
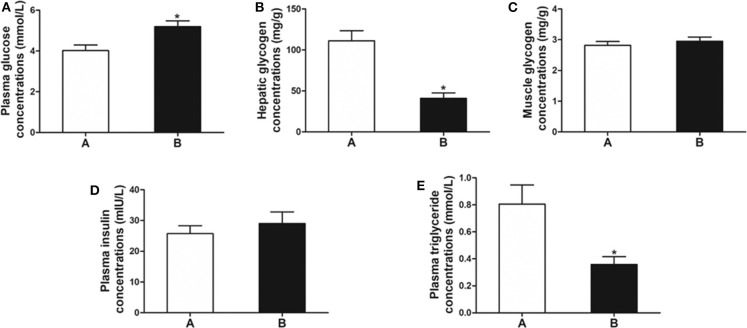
Biochemical indicators related to glucose metabolism. **(A)** Plasma glucose. **(B)** Hepatic glycogen. **(C)** Muscle glycogen. **(D)** Plasma insulin. **(E)** Plasma triglyceride. Data are mean ± SEM (*n* = 6). Significant difference is marked with an asterisk (*P* < 0.05).

The plasma glucose level in fish of Group B was significantly higher than that of Group A (*P* < 0.05) (Figure 1A). The level of hepatic glycogen of Group B was significantly lower than that of Group A (*P* < 0.01) (Figure 1B), whereas there was no difference in muscle glycogen and plasma insulin between the two groups (*P* > 0.05) (Figures 1C,D). In addition, plasma triglyceride was significantly decreased in Group B (*P* < 0.05) (Figure 1E).

### Differentially Expressed Genes and Pathways Based on Transcriptome Sequencing

**Figure 2:**
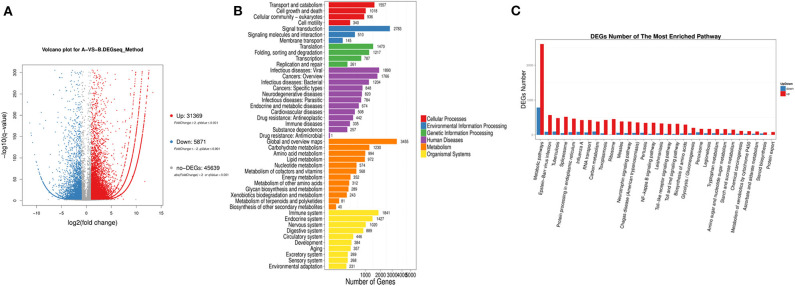
Classification of differentially expressed genes based on transcriptome sequencing. **(A)** Volcano-plot distribution of the differentially expressed gene (red represents the up-regulated gene, blue represents the down-regulated gene, and gray represents the indistinguishable gene). **(B)** Pathway classification map of the differentially expressed genes. **(C)** Up-regulation or down-regulation differentially expressed genes of different pathway.

**Figure 3:**
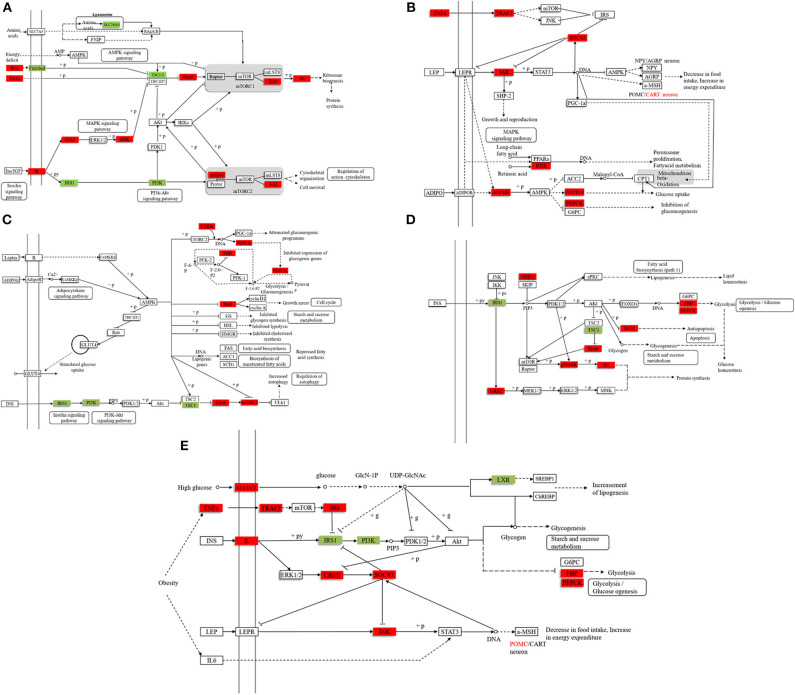
The representative pathways of differentially expressed genes. **(A)** The mTOR signaling pathway. **(B)** The adipocytokine signaling pathway. **(C)** The AMP-activated protein kinase pathway. **(D)** The insulin signaling pathway. **(E)** The glycogen metabolism pathway. Compared with the stable feeding artificial diet group, the red marker indicates that the expression level of the gene is higher, and the green marker indicates that the expression level of the gene is lower. (The filtering condition is FDR ≤ 0.01 and the value of |log2[Ratio]| ≥ 1, and the differentially expressed genes excludes the genes that are not differentially expressed between Group A and C to eliminate the effect of hunger on the mRNA and protein expression levels).

We found 31,369 up-regulated and 5,871 down-regulated genes (Data Sheet 1) to be differentially expressed between Group A and B (Figure 2A), and then the differentially expressed genes were mapped to the reference canonical pathways in KEGG to identify the biological pathways (Figure 2B). To eliminate the effect of nutritional restriction over 5 days on the mRNA and protein expression levels in fish of Group B, we have excluded the genes that did not differentially express between Group A and C from the differentially expressed genes between Group A and B. The representative pathways with the differentially expressed genes were the mTOR signaling pathway, the adipocytokine signaling pathway, the AMP-activated protein kinase pathway, the insulin signaling pathway, and the glycogen metabolism-related pathway (Figure 2C). These genes were involved in the signaling pathways, including the mTOR signaling pathway [*phosphoinositide 3-kinase* (*p13k*), *insulin receptor substrate 1* (*irs1*), *ribosomal protein S6* (*s6*), *Ras homolog, mtorc1 binding* (*Rheb*), and *tuberous sclerosis proteins 1* and 2 (*tsc1/2*)] (Figure 3A); the adipocytokine signaling pathway [*tumor necrosis factor* α (*tnf*α), *janus kinase* (*jak*), *retinoid x receptor*α (*rxr*α), *pepck*, and *glucose transporter member 1/4* (*glut1/4*)] (Figure 3B); the AMP-activated protein kinase pathway [*camp-regulated enhancer b* (*creb*), *pepck, fructose-1, 6-diphosphate* (*fbp*), *irs1, p13k*, and *mtorc1*] (Figure 3C); and the insulin signaling pathway (*irs1, ribosomal s6 kinase* (*s6*k), *growth factor receptor bound protein 2* (*grb2*), *pepck*, and *fbp*) (Figure 3D). In addition, the genes in the glycogen metabolism pathway, including *glut1/2, tnf*α, *tnf receptor-associated factor 2* (*traf2*), *ribosomal s6 kinase* (*s6k*), *camp-regulated enhancer b* (*creb*), *suppressors of cytokine signaling* (*socs3*), *insulin receptor* (*ins-r*), *pepck, fbp, jak*, and *proopiomelanocortin* (*pomc*), were up-regulated in the fish of group B, whereas *insulin receptor substrate 1* (*irs1*) and *phosphoinositide 3-kinase* (*p13k*) genes were decreased in Group B (Figure 3E). The sequencing data in this study have been deposited in the Sequence Read Archive (SRA) database (accession number: SUB6199493 and PRJNA561431).

### P-S6 Expression and *pepck* mRNA Expression

**Figure 4:**
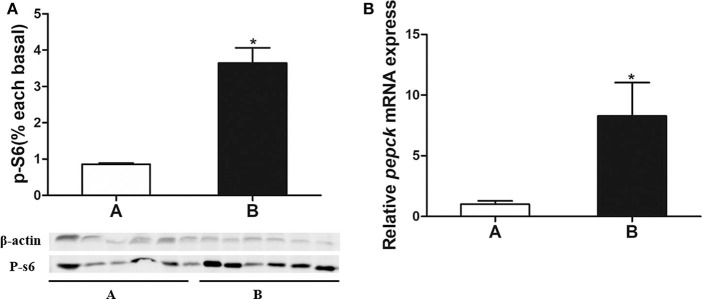
**(A)** Analysis of p-S6 protein expressed in liver of the mandarin fish. **(B)** Validation of *pepck* mRNA expression. Data are mean ± SEM (*n* = 6). Significant difference is marked with an asterisk (*P* < 0.05).

As is shown in Figures 4A,B, the p-S6 protein level and *pepck* mRNA expression were significantly increased in mandarin fish of Group B, which were consistent with the results from transcriptome sequencing.

### *Pepck* Expression Regulated by Histone Methylation

**Figure 5:**
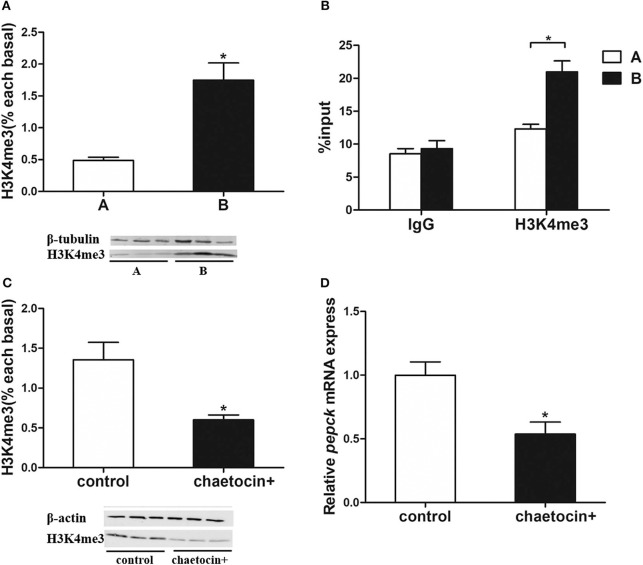
Histone methyltransferases Setd1b increases H3K4me3 level to upregulate *pepck* expression. **(A)** H3K4me3 protein level. **(B)** ChIP assay using IgG control antibody and H3K4me3-specific antibody (*n* = 3). **(C)** Inhibition of Setd1b significantly decreased H3K4me3 level in hepatocytes of mandarin fish. **(D)** Inhibition of Setd1b significantly decreased *pepck* expression in hepatocytes of mandarin fish. Data are mean ± SEM (*n* = 6). Significant difference is marked with an asterisk (*P* < 0.05).

As the target of histone methylation by Setd1b, H3K4me3 protein level was significantly higher in the mandarin fish of Group B than that of Group A (Figure 5A). To investigate the relationship between the methylation of H3K4 and the mRNA expression of *pepck* gene, we conducted a ChIP assay followed by qPCR. The results showed that H3K4me3 had a higher enrichment at the promotor of *pepck* gene in the fish of Group B than that of Group A (Figure 5B).

Then, we used a SETD1B inhibitor chaetocin to treat the hepatocytes of mandarin fish. The western blotting analysis showed that the H3K4me3 protein level was significantly decreased in the hepatocytes with chaetocin treatment (Figure 5C). Furthermore, the real-time quantitative PCR showed that the *pepck* mRNA expression was also diminished with the treatment of SETD1B inhibitor chaetocin (Figure 5D).

## Discussion

Slow growth, high mortality, and low resistance were found when excess carbohydrate ingredients were included in diets (4, 26). Less than 20% of carbohydrate ingredients are suitable for carnivorous fish, such as European plaice (*Pleuronectes platessa)* and salmon (*Oncorhynchus* spp.) (27, 28). In the present study, we observed the anorexia in the mandarin fish fed on the carbohydrate-rich diets (8%). To uncover the molecular mechanism of anorexia in mandarin fish after feeding on carbohydrate-rich diets, we examined the levels of plasma glucose, hepatic glycogen, muscle glycogen, plasma insulin, and plasma triglyceride. The results showed significantly enhanced plasma glucose in the fish with anorexia (Group B) compared with the fish without anorexia (Group A). However, the decreased hepatic glycogen and plasma triglyceride levels were found in the fish of Group B, and the muscle glycogen and plasma insulin levels showed no differences. Previous studies indicate that the levels of plasma glucose, hepatic glycogen, and muscle glycogen increased significantly after feeding fish with high-carbohydrate diets (18, 29). A high-carbohydrate diet induced a higher plasma triglyceride in Wuchang bream (*Megalobrama amblycephala*) (30). It is suggested that the ingested carbohydrates could not be utilized and converted into glycogen and lipid in the mandarin fish with anorexia caused by high-carbohydrate diets, but maintained hyperglycemia. The hyperglycemia with high insulin accounted for a decrease of food intake in mammals after high-carbohydrate feeding (6–8). In mandarin fish, anorexia after feeding high-carbohydrate diets might be attributed to high plasma glucose, which could not be transformed into glycogen or lipid and no enhanced insulin to down-regulate the glucose level.

To investigate the molecular mechanism of the anorexia after high-carbohydrate feeding in mandarin fish, we analyzed the differentially expressed genes between the Group A and B and excluded the genes that did not differentially express between Group A and C. The differentially expressed genes were involved in the mTOR signaling pathway, adipocytokine signaling pathway, AMP-activated protein kinase pathway, and insulin signaling pathway. For the glucose metabolism pathway, the expression of *glut1/2, tnf*α, *traf2, s6k, creb, socs3, ins-r, pepck, fbp, jak*, and *pomc* genes were up-regulated in mandarin fish with anorexia after high-carbohydrate feeding, whereas *irs1* and *p13k* mRNA expression were down-regulated. Previous studies have shown that the leptin signaling pathway could reduce food intake via Jak/STAT pathway by regulation of neuropeptides such as *pomc* (31). Leptin acts through its receptor, which has been shown to recruit Jak, affecting *pomc* expression (32, 33). Our results showed that after feeding high-carbohydrate diets, the *jak* and *pomc* mRNA expressions in the mandarin fish with anorexia was increased, thus inhibiting the food intake.

The activation of the mTOR pathway promotes the translation process and inhibits feeding (34, 35), whereas the inhibition of the mTOR pathway stimulates food intake in rats (36, 37). Our results showed that phosphorylation of the ribosomal protein S6 level was significantly higher in the mandarin fish of Group B, suggesting that the anorexia was related to the activation of mTOR pathway. UDP-N-acetylglucosamine had a negative effect on *irs1* expression (38). After feeding fish carbohydrate-rich diets, carbohydrates could be enzymatically catalyzed to produce UDP-N-acetylglucosamine, which inhibits the expression of *irs1* gene in the mandarin fish with anorexia. In addition, the activation of *mtor* signaling could also inhibit the expression of *irs1* gene in the mandarin fish with anorexia. The high *irs1* expression could facilitate the expression of the key enzyme of gluconeogenesis *fbp* and *pepck*, leading to enhanced gluconeogenesis. Therefore, after feeding the carbohydrate-rich diets, the activation of mTOR-IRS1 pathway could promote gluconeogenesis by the target genes *pepck* and *fbp*, resulting in the high plasma glucose and anorexia. However, what activates the *pepck* expression in the mandarin fish with anorexia was remained to be answered.

To elucidate the regulatory mechanism of *pepck* expression in anorexia, we analyzed the mRNA expression of genes that are involved in the histone methylation from the transcriptome data. We observed that the expression level of *setd1b*, a histone methyltransferase that catalyzes the methylation of H3K4 (17), was up-regulated in the mandarin fish with anorexia. The location of the methyl lysine residue on a histone tail and the degree of methylation (me1, me2, or me3) are associated with the differential gene expression status. H3K4me3 is generally associated with activating chromatin and gene expression (39–41), such as enhancing *p53*-dependent transcription in human colorectal carcinoma HCT116 cells (42), whereas H3K27me3 is associated with repressing the chromatin (43). Previous studies have shown that the modifiers of H3K4me3 play important roles in embryonic development (16) and stem cell biology (44, 45). In the present study, the H3K4me3 level was significantly increased in Group B, which was consistent with the increased *pepck* gene expression in the mandarin fish with anorexia. Previous studies have shown that SETD1B is involved in the tumors and pathology of mammals (17, 46, 47). However, little research has been conducted on its roles in the regulation of glucose metabolism. It was speculated that the methylation of H3K4 mediated by Setd1b might regulate the *pepck* gene expression.

To explore whether H3K4me3 regulates on transcription of *pepck* gene, we examined the interaction between the histone H3K4me3 and the promoter of *pepck* gene with a ChIP assay. We found that the H3K4me3 enrichment at the promoter of *pepck* was significantly increased in the mandarin fish of Group B. Furthermore, we used the SETD1B inhibitor chaetocin to treat the mandarin fish hepatocytes and found the suppressed H3K4me3 protein level, which was accompanied by a significant decrease of *pepck* mRNA expression after chaetocin treatment. In tumor-bearing mice, the inhibition of SETD1B suppressed the H3K4me3 level at the *Nos2* promoter region and diminished the inducible nitric oxide synthase expression in myeloid-derived suppressor cells (17). In mice, a higher enrichment of H3K4me3 at *Fxyd3* gene negatively regulates glucose competence of insulin secreting cells (47), Transcription Factor 19 interacts with H3K4me3 and controls gluconeogenesis by regulating *Glucose-6-phosphatase* and *Fructose-1,6 bisphosphatase* gene expressions (48), suggesting an indispensable epigenetic regulation of H3K4me3 in the glucose metabolism. Our study showed that the increased H3K4me3 modified by Setd1b could enhance the *pepck* mRNA expression to improve gluconeogenesis and thus inhibit the appetite of the mandarin fish with anorexia after feeding carbohydrate-rich diets.

In conclusion, our study indicates a negative effect of carbohydrate-rich diets on the food intake of mandarin fish. The results showed that the ingested carbohydrate could not be converted into lipid and glycogen, but maintains the high plasma glucose, resulting in the reduced food intake and anorexia. The activation of the mTOR and Jak/STAT pathways, and the higher expression levels of *pepck* and *pomc* genes, contributed to the anorexia with the enhanced hyperglycemia. Furthermore, histone methyltransferase Setd1b might tri-methylate the H3K4me3 to increase *pepck* mRNA expression. Our study reported a novel molecular mechanism of appetite regulation after feeding carbohydrate-rich diets.

## Data Availability Statement

The datasets generated for this study can be found in the Sequence Read Archive (SRA) database (accession number: SUB6199493 and PRJNA561431).

## Ethics Statement

The animal study was reviewed and approved by Institutional Animal Care and Use Ethics Committee of Huazhong Agricultural University (Wuhan, China).

## Author Contributions

J-JY and PR contributed to the sample preparation and examination. SH, PR, and X-FL performed the assembly and bioinformatical analysis. Q-QX and Y-PZ performed the data analysis. SH gave technical advice and contributed to the study design. J-JY wrote the paper. All authors read and approved the final manuscript.

## Conflict of Interest

The authors declare that the research was conducted in the absence of any commercial or financial relationships that could be construed as a potential conflict of interest.

## Supplementary Material

Data Sheet 1 The expression profile of the 31,369 up- and 5,871 down-regulated genes.
